# Successful Endourological Management of Encrusted Metallic Ureteral Stents: A Case-Series of Three Patients

**DOI:** 10.3390/reports9030206

**Published:** 2026-06-29

**Authors:** Georgios-Eleftherios Anagnostopoulos, Theodoros Spinos, Vasileios Tatanis, Angelis Peteinaris, Evangelos Liatsikos, Panagiotis Kallidonis

**Affiliations:** 1 Department of Urology, University of Patras Hospital, 26504 Patras, Greece; georgioselef.anagnostopoulos@gmail.com (G.-E.A.); thspinos@otenet.gr (T.S.); tatanisbas@gmail.com (V.T.); peteinarisaggelis@gmail.com (A.P.); liatsikos@yahoo.com (E.L.); 2 Department of Urology, Medical University of Vienna, 1090 Vienna, Austria

**Keywords:** endourology, Wallstent, encrustation, metallic stent, laser management

## Abstract

**Background and Clinical Significance**: Metallic stents represent a breakthrough in the treatment of ureteric obstruction, improving patient quality of life. Despite their advantages, management of encrustation remains a difficult complication to address. This case series highlights the rare occurrence of permanent ureteral Wallstents remaining indwelling for over 20 years. It emphasizes that the function of these older devices can be successfully preserved using minimally invasive techniques. **Case Presentation**: This case series details three patients, two males, aged 75 and 69 years, diagnosed with colon cancer, and one female, aged 67 years, with cervical cancer, who presented with obstructive uropathy due to extrinsic malignant compression. As a therapeutic strategy, permanent ureteral Wallstents were placed in all three patients. Over time, the stents developed significant encrustation, leading to secondary obstruction. Clinical manifestations of this complication varied, ranging from asymptomatic hydronephrosis to acute symptomatic uropathy characterized by fever and localized pain. All cases were treated endoscopically with Ho:YAG laser lithotripsy, and urine flow was successfully restored. During the follow-up period, one patient experienced two recurrences that were managed with the same technique, another remained completely symptom-free, and the third was lost to long-term follow-up. Remarkably, the stents have remained functional for over 20 years post-implantation. **Conclusions**: This is a rare report documenting permanent ureteral Wallstents with such prolonged indwelling time. Furthermore, our findings suggest that through minimally invasive techniques, the function of these devices can be successfully preserved.

## 1. Introduction and Clinical Significance

Ureteric obstruction is a pathological condition in which the urine flow from the renal pelvis to the urinary bladder is obstructed, leading to hydronephrosis and potentially to renal dysfunction and impairment [1]. This condition may manifest due to malignant, iatrogenic, or congenital causes. Early management options include double J stents, percutaneous nephrostomy, or ureteral dilation with a balloon catheter [1]. However, if the obstruction is chronic, a long-term solution includes surgical intervention or minimally invasive options such as metallic stents [1]. The choice of treatment should be individualized for each patient, taking into consideration all the parameters that determine survival and quality of life [1]. There are various types of stents used in the urinary tract, but in the present study, we will focus on metallic stents, and particularly on Wallstents (Boston Scientific, Marlborough, MA, USA). Metallic stents were initially implemented in the vascular and biliary tree and were afterwards used in the urinary tract [2]. Wallstent is the most described and widely used metallic stent in the literature. It is used for benign ureteric obstruction but is especially used for the management of long-term malignant ureteric obstruction [3]. It has also been used for detrusor–sphincter dyssynergia, urethral stricture, and bladder outlet obstruction secondary to Benign Prostatic Hyperplasia (BPH) [4]. Despite their widespread application in the past, complications such as low patency rates, stent migration, encrustation, ingrowth tumor, flank pain, and their difficult removal limited their effectiveness [4]. Encrustation is a very common and difficult-to-manage complication of Wallstents. As a result, newer-generation stents with improved biocompatibility and clinical outcomes have superseded their use [4]. In this study, we present a case series of three patients who developed encrustation on ureteral Wallstents, all of whom were successfully treated endoscopically.

## 2. Case Presentation

This case series presents a triad of patients, two males (75 and 69 years old) and one female (67 years old), with extrinsic malignant ureteral compression. Patients 1 and 2 were diagnosed with colon cancer, while patient 3 was diagnosed with cervical cancer. Initially, to relieve the obstruction, a double J (JJ) ureteral stent was inserted for two weeks in all three patients. Following its removal, a permanent Wallstent was placed in the left ureter in Patients 1 and 3 and in the right ureter in Patient 2. Postoperative monitoring and follow-up Computerized Tomography (CT) urography at one month confirmed appropriate stent function with no evidence of obstruction. At nine years post-insertion, Patient 1 presented with fever, pain, and a serum creatinine level of 1.6 mg/dL due to the obstruction of an encrusted Wallstent. Endoscopic management was performed 1 week after this presentation. Patient 2 presented seven years later with fever and a serum creatinine level of 1.3 mg/dL, undergoing endoscopic intervention 1 month following diagnosis. Patient 3, evaluated at five years post-insertion, was completely asymptomatic; however, significant encrustation, associated hydronephrosis, and a serum creatinine level of 1.3 mg/dL were identified during her clinical evaluation, prompting endoscopic management 1 month later. Encrustation in all three cases was confirmed via KUB X-ray. The baseline, intraoperative, and postoperative characteristics of all patients are summarized in Table 1.

**Figure 1:**
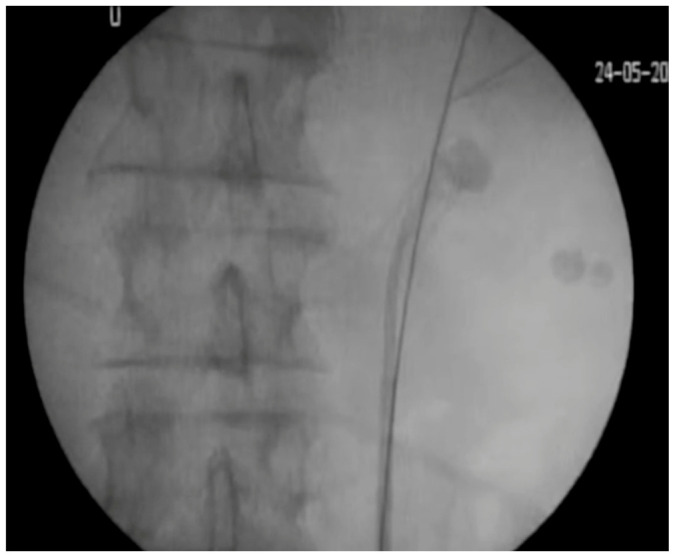
Intraoperative fluoroscopy demonstrating retrograde guidewire advancement into the renal pelvis alongside visible renal calculi.

**Figure 2:**
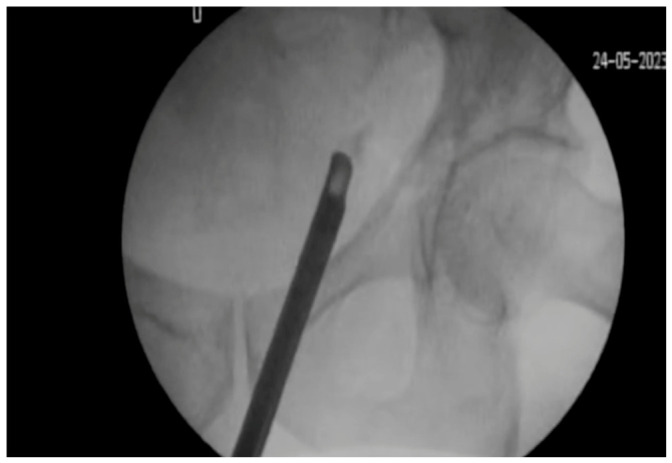
Intraoperative fluoroscopy of the pelvis showing transurethral placement of a rigid cystoscope.

**Figure 3:**
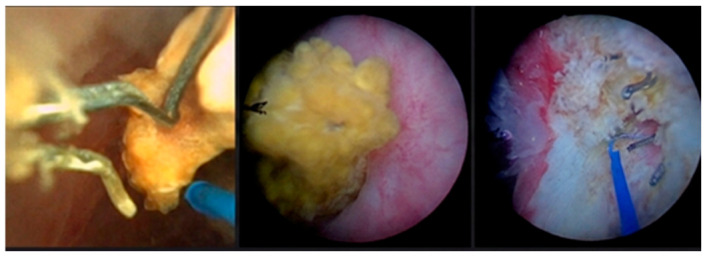
Endoscopic view of an encrusted Wallstent treated with laser lithotripsy.

The endoscopic management technique (laser lithotripsy) was uniform across all three cases. The procedure was performed under general anesthesia in the lithotomy position. Firstly, a cystoscopy was performed using a 22 Fr cystoscope (Karl Storz, Tuttlingen, Germany) to inspect the bladder and evaluate the ureteral orifice. Then, under fluoroscopic guidance, a 0.035-inch hydrophilic guidewire was carefully inserted and advanced into the upper urinary tract (Figure 1), and with the aid of a dual-lumen ureteral catheter, a stiff guidewire was advanced to the pelvicalyceal system. Deployment of a ureteral access sheath (UAS) was neither indicated nor anatomically feasible due to the proximity of the encrusted stent to the ureteral orifice. Instead, an 8/9.8 Fr semi-rigid ureteroscope (Karl Storz, Tuttlingen, Germany) was introduced directly over the stiff guidewire (Figure 2), while the second guidewire was left in place, serving as a safety guidewire. The ureteroscope was advanced gently into the intramural and lower ureter until the encrusted stent was visualized (Figure 3). Then, a flexible ureteroscope was advanced over the Sensor guidewire and lithotripsy was performed, with the stiff guidewire serving as a safety guidewire. In cases where the Wallstent was positioned in close proximity to the ureteral orifice, only a safety guidewire was placed, and the lithotripsy was performed with the semi-rigid ureteroscope. Endoscopic management of the encrustation was achieved utilizing a Holmium:YAG (Ho:YAG) laser device equipped with a 272 μm laser fiber (Quanta, Quanta System, Samarate, Italy). In each case, the Ho:YAG laser was set at 1 J, 8 Hz (8 W). The Virtual Basket mode was used in an attempt to prevent fragment relocation and avoid ureteral wall thermal damage. Residual fragments were removed with the aid of a nitinol basket.

A critical aspect of the surgical technique across all cases was the meticulous application of the laser fiber. In order to avoid any thermal or mechanical damage to the metallic struts of the Wallstents, the fiber was carefully and directly applied exclusively to the calculous encrustation. Strict surgical precision was maintained throughout the procedure. As a result, the structural integrity and long-term functionality of the stents were preserved. At the end of the procedure, a Foley urethral catheter was placed. The urethral catheter was removed on the first postoperative day, and the patient was discharged. Postoperative follow-up at one month revealed excellent clearance of the encrustations. Patient 1 experienced two recurrences of encrustation during his extended follow-up period. The first recurrence was detected five years after the initial treatment, and the second recurrence was detected six years after the first recurrence. Both recurrences were successfully managed using this exact endoscopic laser lithotripsy approach, and his Wallstent has remarkably remained functional for over 20 years post-implantation without further complications. Patient 2 was successfully treated and has remained completely symptom-free. Patient 3 was also managed successfully but was unfortunately lost to long-term follow-up. All patients underwent kidney, ureter, and bladder ultrasonography during the follow-up period, with no evidence of hydronephrosis.

## 3. Discussion

Obstructive uropathy refers to a blockage in the urinary tract, caused by diverse etiologies of malignant, iatrogenic, congenital, or benign origin [5]. It is a complex clinical entity, and if left untreated, it may lead to serious complications such as hydronephrosis, progressive renal failure, urinary tract infection and sepsis. This condition may start as an acute episode and progress to a chronic disease. Symptoms may present as flank pain, hematuria and lower urinary tract symptoms [5]. The diagnostic protocol consists of clinical assessment, laboratory testing, imaging evaluation, and urine analysis. Specifically, renal function is monitored by measuring creatinine and BUN levels. Regarding imaging testing, ultrasound (U/S) and computed tomography (CT) are the primary diagnostic tools. Alternative tests, such as intravenous pyelogram, voiding cystourethrogram, renal nuclear scans, and MRI, can be used under specific circumstances [5].

Management of obstructive uropathy focuses on the decompression of the urinary system [6]. Treatment is different between lower and upper urinary tract disease. In cases of lower urinary tract obstruction, immediate drainage is required through urethral or suprapubic catheterization [6]. In contrast, regarding upper urinary tract blockage, drainage is achieved using either JJ stents or via percutaneous nephrostomy. In relation to malignant obstructive uropathy, a preferable choice of treatment is metallic stents, which represents a permanent solution [6].

The innovation of metallic stents revolutionized the minimally invasive approaches in the field of endourology [2]. At first, they were used in the vascular and biliary tree and subsequently adopted by urologists. The Wallstent, composed of braided cobalt-based alloy monofilament wires, was the first metallic stent inserted in the ureter [2]. These devices were proposed as an alternative to polymeric stents in the management of malignant obstructive uropathy. In chronic cases where long-term drainage is required, metallic stents are superior due to resistance to external compression, long-term patency, and reduced need for frequent exchanges [3]. Despite these advantages and widespread application, complications have been reported, including flank pain, stent migration, tumor ingrowth, hyperplastic reaction and encrustation. In particular, the combination of encrustation and difficult removal of the stent is a significant challenge for endourologists [3].

When a stent is placed for too long inside the ureter, mineral crystals can build up on or inside it, a process called encrustation. These crystals eventually form stones that can block the urinary tract and damage surrounding tissue [7]. In an attempt to address these complications, Tomer et al. proposed an algorithm. In mild cases, with stones under 5 mm and covering less than half the stent surface, cystoscopic removal is generally sufficient. For severe cases, with crystals larger than 5 mm and/or covering more than half of the stent surface, the treatment depends on the location of encrustation. For stones at the proximal (renal) end of the stent, measuring less than 1.5 cm, treatment is achieved using extracorporeal shock wave lithotripsy (ESWL) [7]. In contrast, stones with diameters of 1.5 cm or larger are managed with percutaneous nephrolithotomy (PCNL). When the stones are formed at the stent shaft, ureteroscopy with laser lithotripsy is considered the treatment of choice. Finally, when stones are located at the distal (bladder) end of the stent, cystolitholapaxy is selected as a typical treatment. If minimally invasive approaches fail, open or laparoscopic procedures may be necessary [7].

All the available data about the Wallstent were evaluated in a systematic review by Kallidonis et al. [4]. The results demonstrated that the role of the Wallstent in the management of malignant obstructive uropathy was insufficient. These devices, in contrast to double-J polymeric stents, showed improved patency rates, prolonged indwelling times, and reduced stent-related complications. Although these metallic stents had several advantages, complications, and especially low long-term patency rates, led to their replacement. In an ongoing effort to minimize stent morbidity, different types of metallic stents have been introduced over the years, including self-expandable, thermo-expandable, balloon-expandable, and non-expandable/coiled types [4,8]. To evaluate all the new devices, Bian et al. conducted a systematic review and meta-analysis comparing various metallic stents. The study concluded that covered stents were superior compared to uncovered ones, with Allium stents being the most optimized option [9].

This case series presents a triad of patients with permanent metallic stents (Wallstents) who developed encrustation on the stent surface. There is plenty of information in the literature regarding the management of encrusted polymeric stents. Pais et al., in a multicenter study, proposed percutaneous nephrolithotomy as the preferred treatment for encrusted polymeric stents [10]. Regarding metallic stents, there are a few cases addressing the management of encrustation and its removal. None of these cases concerned the Wallstent. In particular, Kawahara et al. reported a case in which an encrusted metal alloy (MP35N) stent was managed with Ho:YAG laser lithotripsy [11]. Pavlinec et al. also used Ho:YAG laser lithotripsy to treat the stones along the stent (Resonance, Cook Medical LLC., Bloomington, IN, USA), and pioneered a percutaneous removal technique using a guidewire loop [12]. Furthermore, Altyeb et al. performed robotic surgery and pyeloplasty for an encrusted metallic stent (Memokath) [13]. Given the permanent nature of metallic stents in oncology patients, a structured and lifelong follow-up protocol is imperative to prevent silent renal failure caused by recurrent encrustation or tumor progression. At our institution, cancer patients with indwelling metallic stents undergo routine clinical and laboratory evaluations every 3 to 6 months, focusing on serum creatinine and blood urea nitrogen (BUN) levels to monitor renal function. Concurrently, renal ultrasonography is performed at each visit to screen for the development of new or worsening hydronephrosis. Surveillance computed tomography (CT) or magnetic resonance imaging (MRI) is not routinely scheduled for stent monitoring alone but is performed in accordance with the patient’s oncological staging, or immediately if acute symptomatic uropathy is suspected.

In the present study, all patients were successfully treated endoscopically with Ho:YAG laser lithotripsy. Notably, for Patients 1 and 2, the Wallstents have remained functional for over two decades post-implantation, while the ultimate long-term functionality for Patient 3 remains unknown due to her loss to follow-up. This evidence supports the potential long-term durability of the Wallstent in selected patients. Furthermore, our experience suggests that endoscopic management of encrustation is feasible and effective, allowing preservation of the stent and continued function. To our knowledge, this is a unique report documenting permanent ureteral Wallstents with such prolonged indwelling time and high patency.

### Limitations

This study has several limitations that must be acknowledged. First, the retrospective, single-center design introduces potential selection bias and relies on the accuracy of historical medical records. Second, our study consists of a small sample size without a comparator or control group, which limits the statistical enhancement of our outcomes. Finally, biochemical stone analysis of the encrustations was not performed [14,15,16]. The lack of stone analysis is a notable limitation. Identifying the specific composition could have guided targeted prophylactic medical management and potentially reduced the risk of the recurrences observed, particularly in Patient 1.

## 5. Conclusions

Metallic stent encrustation remains a significant challenge for endourologists, especially when older-generation metallic stents (i.e., Wallstents) are involved. Laser lithotripsy may be considered as a feasible, safe, and efficient therapeutic strategy. Our case series presents a rare triad of patients with extrinsic obstructive uropathy and encrusted Wallstents who were successfully managed with an endourological approach (laser lithotripsy). In complex or recurrent cases, repeated treatment sessions may be required.

## Figures and Tables

**Table 1:** Baseline, intraoperative and postoperative characteristics of patients undergoing endourological management of encrusted Wallstents.

Patient	Patient 1	Patient 2	Patient 3
Age (years-old)	75	69	67
Sex	Male	Male	Female
Side	Left	Right	Left
Etiology	Colon Cancer	Colon Cancer	Cervical Cancer
Symptoms	Obstructive Uropathy (Fever and Pain)	Obstructive Uropathy (Fever)	No
Recurrence	Yes (2 times)	No	N/A (lost follow-up)
Management	Laser Lithotripsy	Laser Lithotripsy	Laser Lithotripsy
Laser Type	Holmium:YAG	Holmium:YAG	Holmium:YAG
Settings	1 J, 8 Hz (8 W)	1 J, 8 Hz (8 W)	1 J, 8 Hz (8 W)
Follow-up	2 recurrences (both successfully managed with laser lithotripsy)	Symptoms-free	Patient has lost follow-up

## Data Availability

The original contributions presented in this study are included in the article. Further inquiries can be directed to the corresponding author.

## Abbreviations

The following abbreviations are used in this manuscript:

BPH	Benign Prostatic Hyperplasia
JJ	Double-J
BUN	Blood Urea Nitrogen
CT	Computed Tomography
ESWL	Extracorporeal Shock Wave Lithotripsy
Ho:YAG	Holmium:YAG (Yttrium Aluminum Garnet)
Hz	Hertz
J	Joules
MRI	Magnetic Resonance Imaging
PCNL	Percutaneous Nephrolithotomy
U/S	Ultrasound
UAS	Ureteral Access Sheath
W	Watts
μm	Micrometers
Fr	French

## References

[1] Hori T., Makino T., Kawahara T., Urata S., Miyagi T. (2023). Effectiveness of Double-J Metallic Mesh Ureteral Stents for Malignant Ureteral Obstruction: A Retrospective Study. Vivo.

[2] Arguinarena F.J., del Busto E.F. (2004). Self-expanding polytetrafluoroethylene covered nitinol stents for the treatment of ureteral stenosis: Preliminary report. J. Urol..

[3] Liatsikos E.N., Karnabatidis D., Katsanos K., Kallidonis P., Katsakiori P., Kagadis G.C., Christeas N., Papathanassiou Z., Perimenis P., Siablis D. (2009). Ureteral metal stents: 10-year experience with malignant ureteral obstruction treatment. J. Urol..

[4] Kallidonis P., Kotsiris D., Sanguedolce F., Ntasiotis P., Liatsikos E., Papatsoris A. (2017). The effectiveness of ureteric metal stents in malignant ureteric obstructions: A systematic review. Arab. J. Urol..

[5] Rishor-Olney C.R., Hinson M.R. (2023). Obstructive Uropathy. StatPearls.

[6] Pérez-Aizpurua X., Cabello Benavente R., Bueno Serrano G., Alcázar Peral J.M., Gómez-Jordana Mañas B., Tufet I Jaumot J., Ruiz de Castroviejo Blanco J., Osorio Ospina F., Gonzalez-Enguita C. (2024). Obstructive uropathy: Overview of the pathogenesis, etiology and management of a prevalent cause of acute kidney injury. World J. Nephrol..

[7] Tomer N., Garden E., Small A., Palese M. (2021). Ureteral Stent Encrustation: Epidemiology, Pathophysiology, Management and Current Technology. J. Urol..

[8] Sali G.M., Joshi H.B. (2020). Ureteric stents: Overview of current clinical applications and economic implications. Int. J. Urol..

[9] Bian X., Hu H., Tian C., Wang C., Lai C.H., Wang M., Ji J., Xu K., Xu T., Hu H. (2025). Comparison of different segmental metal ureteral stents as maintenance therapy across different years in ureteral stricture management: A systematic review and meta-analysis. Int. J. Surg..

[10] Pais V.M., Chew B., Shaw O., Hyams E.S., Matlaga B., Venkatesh R., Page J., Paterson R.F., Arsovska O., Kurtz M. (2014). Percutaneous nephrolithotomy for removal of encrusted ureteral stents: A multicenter study. J. Endourol..

[11] Kawahara T., Kobayashi K., Kurora S., Yao M., Uemura H. (2020). Successful removal of an encrusted metallic ureteral stent using a disposable ureteroscope and Ho:YAG laser lithotripsy. Urolithiasis.

[12] Pavlinec J.G., Rabley A.K., Gordon A.O., Kuo J., Bird V.G. (2020). Percutaneous Removal of Retained Metallic Ureteral Stent with a Looped Polytetrafluoroethylene-Coated Guidewire. J. Endourol. Case Rep..

[13] Altyeb A.B., Khalil I.A., Tawfik H., Okasha N., Abomarzouk O., Ansari A.A. (2025). Early encrustation of a ureteric metallic stent managed by robotic assisted extraction and pyeloplasty. Radiol. Case Rep..

[14] Vanderbrink B.A., Rastinehad A.R., Ost M.C., Smith A.D. (2008). Encrusted urinary stents: Evaluation and endourologic management. J. Endourol..

[15] Rouprêt M., Daudon M., Hupertan V., Gattegno B., Thibault P., Traxer O. (2005). Can ureteral stent encrustation analysis predict urinary stone composition?. Urology.

[16] Hu J., Zhao W., Xiao G., Gao F., Peng M., Huang R., Ouyang K., Qiu H., Zhong B., Wang L. (2026). Quantitative analysis and risk factors of high-grade encrustation in double-J stents after ureteroscopy and laser lithotripsy. Urolithiasis.

